# A Comparative Study on the Influence of Zirconia and Titanium Nanoparticles on the Functionality of Osteocytes In Vitro

**DOI:** 10.1155/ijod/6486868

**Published:** 2026-06-14

**Authors:** Mustafa Hayder Matouk, K. G. Aghila Rani, Waad Kheder, Asmaa Ismail, A. R. Samsudin, Sausan AlKawas

**Affiliations:** ^1^ Department of Oral and Craniofacial Health Sciences, College of Dental Medicine, University of Sharjah, Sharjah, UAE, sharjah.ac.ae; ^2^ Research Institute for Medical and Health Sciences, University of Sharjah, Sharjah, UAE, sharjah.ac.ae; ^3^ Restorative and Preventive Dentistry Department, College of Dental Medicine, University of Sharjah, Sharjah, UAE, sharjah.ac.ae

**Keywords:** bone remodeling, osteocytes sclerostin, titanium-dioxide nanoparticles, zirconia-dioxide nanoparticles

## Abstract

**Introduction and Aim:**

The aim of this study was to compare the influence of zirconia dioxide nanoparticles (ZrO_2_‐NPs) and titanium dioxide nanoparticles (TiO_2_‐NPs) on the functionality of osteocytes in vitro.

**Methods:**

MLO‐Y4 osteocytic cells were treated with varying concentrations of ZrO_2_‐NPs or TiO_2_‐NPs for viability studies. Apoptosis assays following treatment with 100 and 500 µg/mL of ZrO_2_‐NPs or TiO_2_‐NPs for 72 h were performed. Sclerostin (SOST) levels were assessed at 24 and 72 h, while receptor activator of nuclear factor kappa‐B ligand (RANKL) and osteoprotegerin (OPG) were measured at 72 h by ELISA and real‐time PCR analysis.

**Results:**

Viability assays revealed a dose‐dependent cytotoxicity for both types of nanoparticles, with TiO_2_‐NPs reducing cell viability at 100 µg/mL within 24 h, while ZrO_2_‐NPs promoted proliferation at lower concentrations but showed similar cytotoxicity at higher doses. Apoptosis and necrosis assays revealed a dose‐dependent cytotoxic response, with TiO_2_‐NPs inducing significant cell death at 100 µg/mL, while ZrO_2_ NPs showed minimal effects at the same concentration; at 500 µg/mL, both nanoparticles markedly increased apoptosis, with TiO_2_‐NPs eliciting higher apoptosis. *SOST* gene expression and SOST release were significantly enhanced following exposure to both ZrO_2_‐NPs and TiO_2_‐NPs, with TiO_2_‐NPs inducing significantly higher levels. TiO_2_‐NPs upregulated RANKL while downregulating OPG both at the gene and protein levels. Although ZrO_2_‐NPs exhibited a similar trend, their impact on *SOST* expression, RANKL, and OPG release was notably lower, suggesting a potentially less disruptive impact on bone remodeling.

**Conclusion:**

The findings offer valuable insights into the osteocyte‐mediated effects of implant‐related nanoparticles. ZrO_2_‐NPs are a less disruptive alternative to TiO_2_‐NPs for bone implants, offering insights into osteocyte‐mediated remodeling and guiding biomaterial selection for implant longevity.

## 1. Introduction

Endosseous titanium dental implants are widely regarded as a highly effective treatment in oral and maxillofacial prosthetic rehabilitation, showing a high 10‐year survival rate [1]. Despite these success rates, long‐term challenges leading to implant failures can occur due to factors such as biomechanical instability combined with biofilm‐induced infections [2], resulting in peri‐implantitis and bone loss [3, 4]. Recently, tribocorrosion and the release of particles from the implant surface [5] were observed as an additional multifactorial disease factor driven by bacterial infection and mechanical stress. Particulate debris has been detected in the peri‐implant soft tissues [6] as well as in the mineralized bone adjacent to the implants [7]. The release of titanium micro‐ and nanoparticles into the peri‐implant microenvironment contributes significantly to peri‐implantitis, triggering foreign body reactions and elevating proinflammatory cytokine levels, which in turn promote bone and soft tissue loss [8]. Other workers have observed the differential effects between microparticles and nanoparticles on the peri‐implant cells and advocated microparticles to cause more harm to bone and soft tissue regeneration compared to that of nanoparticles [9].

The impact of tribocorrosion has demanded a search for more compatible implant materials that produce less harmful degradation particles following corrosion. In this context, zirconia (ZrO_2_) nanoparticles have emerged as a promising alternative due to their excellent physical, biological, esthetic, and corrosion‐resistant properties. ZrO_2_‐based implants are now the second most commonly used dental implants, with success rates comparable to those of titanium implants, close to 95% at 5 years [10, 11]. Recent long‑term clinical data further support the material stability of ZrO_2_ implants, with reports of favorable 5‑year outcomes and low degradation‐related complications, underscoring their relevance as a corrosion‑resistant alternative [11].

Although zirconia and titanium are commonly used in orthopedic and dental implants, implant abutments, and ceramic restorations [12], zirconia implant surfaces are reported to release significantly lower amounts of ions into peri‐implant tissues than titanium [13]. This difference in degradation behavior is clinically relevant as tribocorrosion‐driven nanoparticle release is increasingly recognized as a contributor to peri‑implant inflammation and bone loss. Research has increasingly focused on the cytotoxic effects of both ZrO_2_‐NPs and TiO_2_‐NPs, highlighting their internalization by cells and subsequent impact on cell viability and function [14]. High concentrations of these nanoparticles have been shown to induce oxidative stress and cytotoxicity [15]. While most workers have focused on the impact of these particles on osteoblasts and osteoclasts, which are considered to be the key bone regenerative cells, their effects on osteocytes are less well understood. Recent studies have shown that osteocytes are the main orchestrator of the bone remodeling process [16]. Their “trapped location” around mineralized tissue may confer protection against a toxic environment, but recent investigations have shown upregulated expression of catabolic markers associated with osteocytic osteolysis in response to unrecognized action of wear particles on osteocytes from orthopedic materials [17].

The aim of this study was to compare the influence of zirconia dioxide nanoparticles (ZrO_2_‐NPs) and titanium dioxide nanoparticles (TiO_2_‐NPs) on osteocytes functionality in vitro. Although several studies have examined the effects of TiO_2_‐ and ZrO_2_‐NPs on osteoblasts and osteoclasts, data on osteocyte responses, particularly in MLO‑Y4 cells, remain scarce. To our knowledge, no prior study has directly compared TiO_2_‑NPs and ZrO_2_‑NPs in osteocytes, despite their central regulatory role in bone remodeling.

## 2. Materials and Methods

### 2.1. Cell Culture

Murine MLO‐Y4 cells (AddexBio, USA) were cultured in alpha‐minimum essential medium (α‐MEM; Invitrogen, USA) containing 10% FBS (Sigma Aldrich, USA) and 1% antibiotics (Sigma) and maintained at 37°C in a humidified incubator containing 5% CO_2_. MLO‐Y4 osteocyte‐like cells were selected as an established model for osteocyte biology because they display a dendritic morphology, osteocyte‐like marker profile, and the capacity to regulate osteoclastogenesis and osteoblast function and are widely used to study osteocyte responses to mechanical and material stimuli in vitro [18].

### 2.2. Preparation of ZrO_2_‐NPs and TiO_2_‐NPs

The hydrodynamic diameter, polydispersity index (PDI), and the surface charge of TiO_2_‐NPs were analyzed using a Malvern Zetasizer Nano‐ZS system (Malvern Instruments, United Kingdom) as previously described in our earlier study (Table S1) [19]. ZrO_2_‐NPs and TiO_2_‐NPs (both Sigma) with a diameter of less than 100 nm were UV‐sterilized for 1 h and prepared in sterile Dulbecco’s phosphate‐buffered saline (DPBS) at a final concentration of 5 mg/mL. Solutions were then sonicated for 1 min using a Qsonica Q125‐220 Sonicator (USA). TiO_2_‐NPs served as the positive control in this study, while untreated cells were used as the negative control.

To ensure comparable colloidal behavior during cell exposure, both ZrO_2_‑NPs and TiO_2_‑NPs were dispersed using identical preparation conditions, including the same sonication protocol, solvent (DPBS), pH, and serum‑free environment. The ZrO_2_‑NPs used in this study were supplied as commercially precharacterized materials, with manufacturer‑verified specifications indicating a primary particle size of <100 nm, spherical morphology, and >99% purity. Although full hydrodynamic characterization (size distribution, PDI, and zeta potential) was not performed for ZrO_2_‑NPs in the present study, the use of identical dispersion conditions for both nanoparticle types minimizes variability in agglomeration behavior during in vitro exposure.

### 2.3. Cell Viability

MLO‐Y4 cells were seeded in triplicate into 96‐well tissue culture‐treated plates at a density of 5 × 10^3^ cells/well in 100 µL of complete α‐MEM. Following 24 h of incubation, the cells were exposed to 25, 50, 100, 500, and 1000 µg/mL of ZrO_2_‐NPs or TiO_2_‐NPs at different time points such as 24, 48, and 72 h. Representative images of treated cells at all concentrations were acquired using an Olympus microscope (Olympus Life Science, Tokyo, Japan). Cell viability was subsequently assessed using the XTT assay following the manufacturer’s protocol (Cell Proliferation Kit II, Roche, United Kingdom). Briefly, after removal of the treatment media, cells were incubated with the XTT reagent. The optical density was then measured at 450 nm using a BioTek 800 TS microplate reader. To minimize potential interference caused by nanoparticle‐associated light scattering or absorbance, blank wells containing culture medium, nanoparticles, and the XTT reagent without cells were included in parallel. The absorbance values obtained from these nanoparticle blanks were subtracted from the corresponding experimental readings prior to viability analysis.

### 2.4. Measurement of Cellular Apoptosis/Necrosis

Cells were seeded at 2.5 × 10^5^ seeding density in 6 well plates in duplicates and incubated for 24 h in a humidified chamber. Cells were then treated with 100 and 500 µg/mL of ZrO_2_‐NPs and TiO_2_‐NPs for 72 h. After 72 h of treatment, cells were trypsinized and subjected to an apoptosis assay using the Annexin V apoptosis detection kit FITC (ThermoFisher Scientific, USA) following the manufacturer’s protocol. The results were analyzed using a BD FACS Aria III cell sorter (Becton Dickinson Biosciences, UK). Debris was excluded based on FSC/SSC characteristics, and doublets were removed using FSC‑A vs. FSC‑H gating. Unstained cells and untreated control cells were used to define the negative population and establish quadrant boundaries for Annexin V and PI. A minimum of 10,000 singlet events were collected per sample. Flow cytometry results were then analyzed using the FlowJo software.

### 2.5. ELISA Assays

Sclerostin (SOST) levels were analyzed at both 24 and 72 h using ELISA, while receptor activator of nuclear factor kappa‐B ligand (RANKL) and osteoprotegerin (OPG) levels were measured only at 72 h, following the manufacturers’ protocols. Briefly, cell culture supernatants from MLO‐Y4 cells grown in six‐well plates were collected after treatment with 500 µg/mL ZrO_2_‐NPs and TiO_2_‐NPs and used to detect the amount of mouse SOST (AB213889, Abcam, USA), RANKL (RK00341, Abclonal, USA), and OPG (AB100617, Abcam, USA) following the manufacturer’s protocol.

### 2.6. Real‐Time PCR Analysis

MLO‐Y4 cells were seeded in six‐well plates in triplicate at 2.5 × 10^5^ seeding density and incubated for 24 h. Cells were then treated with 500 µg/mL ZrO_2_‐NPs and TiO_2_‐NPs for 72 h. After 72 h, cells were washed with DPBS, trypsinized, and collected for RNA extraction using the RNeasy Protect Mini Kit (Qiagen, Germany). A 500 ng of RNA from each sample was then converted into cDNA using the QuantiTect Reverse Transcription Kit (Qiagen, Germany), following the manufacturer’s protocol. Real‐time PCR using HOT FIREPol EvaGreen qPCR Supermix (Solis Biodyne Tartu, Estonia) was performed in a StepOne Real‐Time PCR machine (ThermoFisher Scientific, USA) using primers mentioned in Table 1. Relative mRNA expression was quantified using the comparative 2^−ΔΔCt^ method, with 18S as the reference gene and untreated controls as the calibrator. Primer specificity and single‐product amplification were confirmed by melt curve analysis, showing single peaks for all primer pairs.

**Table 1:** Primer sequences, annealing temperatures, and amplicon sizes of genes used in quantitative real‐time PCR (qPCR) analysis.

Gene	Primer sequence (forward)	Primer sequence (reverse)	Annealing temp (°C)	Amplicon size (bp)
*18S*	5′‐GGAGAGGGAGCCTGAGAAAC‐3′	5′‐CCTCCAATGGATCCTCGTTA‐3′	60	~171
*SOST*	5′‐AAGCCTTCAGGAATGATGCCA‐3′	5′‐GAGGTCTGCCTCCATTCTCC‐3′	60	~111
*OPG*	5′‐TGTTCCGGAAACAGAGAAGC‐3′	5′‐CTCTCGGCATTCACTTTGGT‐3′	60	~155
*RANKL*	5′‐GCGCTTCTCAGGAGCTCC‐3′	5′‐CATTGATGGTGAGGTGTGCA‐3′	60	~101

### 2.7. Statistical Analyses

For all experiments, significance was determined using one‐way ANOVA with Tukey’s post hoc pairwise comparisons. As for SOST ELISA and cell viability, the significance was determined using two‐way ANOVA and Bonferroni post hoc pairwise comparisons. Normality was assessed using the Shapiro–Wilk test, and the homogeneity of variances was verified using Levene’s test. All datasets met the assumptions for ANOVA, and no data transformations were required. *p* values < 0.05 were considered significant. All quantitative data represent the mean ± SD of triplicates of independent experiments, and all graphs were created using the GraphPad Prism Version 9.1.0 software (GraphPad Software, USA).

## 3. Results

### 3.1. Osteocyte Morphology and Viability

**Figure 1:**
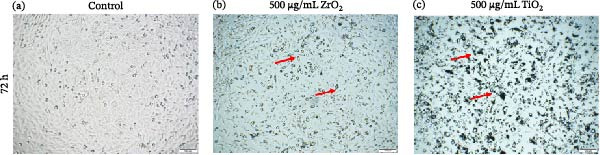
Attachment of MLO‐Y4 osteocytic cells following 72 h exposure to 500 µg/mL ZrO_2_‐NPs and TiO_2_‐NPs, with untreated cells as control. Representative microscopy images showing (a) control MLO‐Y4 cells, (b) MLO‐Y4 cells treated with 500 µg/mL ZrO_2_‐NPs, and (c) TiO_2_‐NPs at 72 h of culture. Scale bar = 100 µm for all images. Magnification = 100 µm.

MLO‐Y4 cells treated with both ZrO_2_ and TiO_2_ NPs at 500 µg/mL, along with the untreated control, are shown in Figure 1. At the concentrations of 500 and 1000 µg/mL, cells presented a round morphology, with a loss of their stellate morphology.

**Figure 2:**
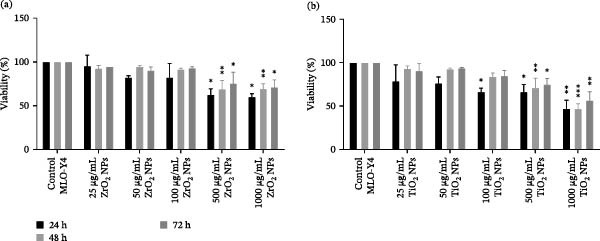
Histograms representing cell viability of MLO‐Y4 cells posttreatment with ZrO_2_‐NPs and TiO_2_‐NPs. (a) Cell viability at 24, 48, and 72 h posttreatment with (A) ZrO_2_‐NPs and (b) TiO_2_‐NPs. For ZrO_2_‐NPs, cells showed a significant decrease in viability starting at a 500 µg/mL concentration. For TiO_2_‐NPs, cells showed a significant decrease in viability starting at a 100 µg/mL concentration. Data represent the mean ± SD of three (*n* = 3) biological replicates ( ^∗^ *p* < 0.05,  ^∗∗^ *p* < 0.01,  ^∗∗∗^ *p* < 0.001).

Cell viability studies revealed a dose‐ and time‐dependent response of MLO‐Y4 osteocytes upon treatment with ZrO_2_‐NPs and TiO_2_‐NPs. For ZrO_2_‐NPs, a time‐dependent increase in cellular proliferation was seen at lower concentrations, such as 25, 50, and 100 µg/mL concentrations, whereas significant reductions in cell viability were observed at 500 and 1000 µg/mL across all time points (Figure 2a). In the case of TiO_2_‐NPs, no significant reduction in cell viability was observed at any time points for lower concentrations such as 25 and 50 µg/mL. However, at 100 µg/mL, a statistically significant reduction in cell viability was noted at the 24‐h time point compared to that of the untreated control cells. As the concentrations further increased to 500 and 1000 µg/mL, cell viability significantly declined compared to that of the untreated control cells (Figure 2b).

### 3.2. Cellular Apoptosis and Necrosis

**Figure 3:**
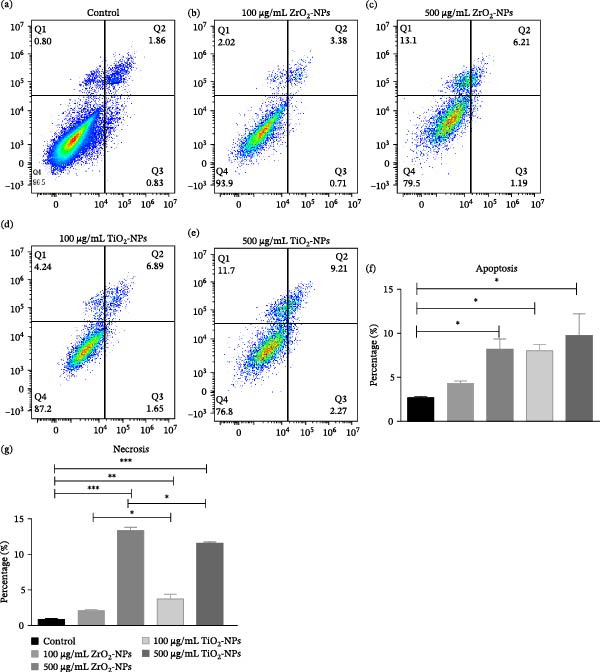
Flow cytometry analysis of cellular apoptosis and necrosis posttreatment of MLO‐Y4 cells with ZrO_2_‐NPs or TiO_2_‐NPs for 72 h. Representative dot plots of MLO‐Y4 cells treated with (a) complete culture media (control), (b) 100 µg/mL, (c) 500 µg/mL ZrO_2_‐NPs, (d) 100 µg/mL, and (e) 500 µg/mL TiO_2_‐NPs. (f) Histogram representing the total percentage of apoptosis of MLO‐Y4 cells treated with 100 and 500 µg/mL of ZrO_2_‐NP_s_ or TiO_2_‐NPs. (g) Histogram representing the total percentage of necrosis of MLO‐Y4 cells treated with 100 and 500 µg/mL of ZrO_2_‐NPs or TiO_2_‐NPs. Data represent the mean ± SD of three (*n* = 3) biological replicates ( ^∗^ *p* < 0.05,  ^∗∗^ *p* < 0.01,  ^∗∗∗^ *p* < 0.001).

Treatment of MLO‐Y4 cells with 100 µg/mL ZrO_2_‐NPs showed no significant difference in cellular apoptosis and necrosis compared to the control group (Figure 3a,b). However, at 500 µg/mL ZrO_2_‐NPs (Figure 3c), 100 µg/mL TiO_2_‐NPs (Figure 3d), and 500 µg/mL TiO_2_‐NPs (Figure 3e) concentrations, a significant increase in cellular apoptosis was observed (*p* < 0.05), with 500 µg/mL TiO_2_‐NPs being the highest (Figure 3f). Likewise, a significant difference in necrosis among the control and cells treated with 500 µg/mL ZrO_2_‐NPs (*p* < 0.001), 100 µg/mL TiO_2_ (*p* < 0.01) and 500 µg/mL TiO_2_‐NPs (*p* < 0.001) (Figure 3g). Notably, cells treated with ZrO_2_‐NPs showed significantly lower cellular necrosis than those treated with TiO_2_‐NPs (*p* < 0.05). However, at 500 µg/mL concentration, cells treated with ZrO_2_‐NPs showed a significantly higher cellular necrosis than TiO_2_‐NPs (*p* < 0.05) (Figure 3c,g).

### 3.3. *SOST* Expression and Release by MLO‐Y4 Osteocytes Exposed to ZrO_2_‐NPs and TiO_2_‐NPs

**Figure 4:**
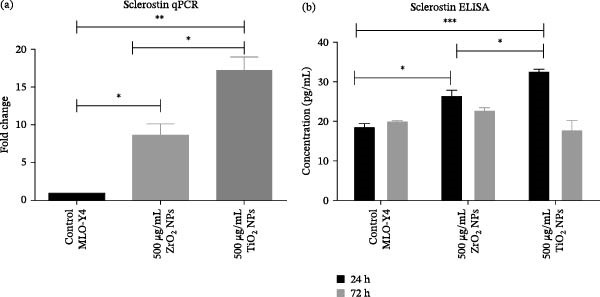
Expression of sclerostin (*SOST*) gene and protein by qPCR and ELISA. (a) Histogram representing qPCR quantification of the total SOST gene by MLO‐Y4 cells treated with 500 µg/mL ZrO_2_‐NPs and TiO_2_‐NPs at 72 h. (b) Histogram representing ELISA measurement of the total SOST protein release by MLO‐Y4 cells treated with 500 µg/mL ZrO_2_‐NPs and TiO_2_‐NPs at 24 and 72 h. Data represent the mean ± SD of three (*n* = 3) biological replicates ( ^∗^ *p* < 0.05,  ^∗∗^ *p* < 0.01,  ^∗∗∗^ *p* < 0.001).

In comparison to the control group, *SOST* expression was significantly upregulated in MLO‐Y4 cells following treatment with both 500 µg/mL ZrO_2_‐NPs (8.63‐fold ± 1.06; *p* < 0.05) and 500 µg/mL TiO_2_‐NPs (17.22‐fold ± 1.22; *p* < 0.001). Notably, TiO_2_‐NPs induced significantly higher SOST mRNA expression than ZrO_2_‐NPs (*p* < 0.05; Figure 4a).

Whereas SOST protein release increased significantly after 24 h of treatment with 500 µg/mL ZrO_2_‐NPs (26.58 ± 1.27 pg/mL; *p* < 0.05) and 500 µg/mL TiO_2_‐NPs (32.43 ± 0.719735 pg/mL; *p* < 0.001) compared to the control (18.43 ± 0.99 pg/mL), consistent with gene expression. TiO_2_‐NPs also induced greater SOST release than ZrO_2_‐NPs (*p* < 0.05). However, by 72 h, SOST protein levels in the supernatant returned to baseline with no significant differences across all groups, suggesting potential protein degradation or rapid turnover despite sustained mRNA upregulation at this time point (Figure 4b).

### 3.4. *RANKL* Expression and Release by MLO‐Y4 Osteocytes Exposed to ZrO_2_ and TiO_2_‐NPs

**Figure 5:**
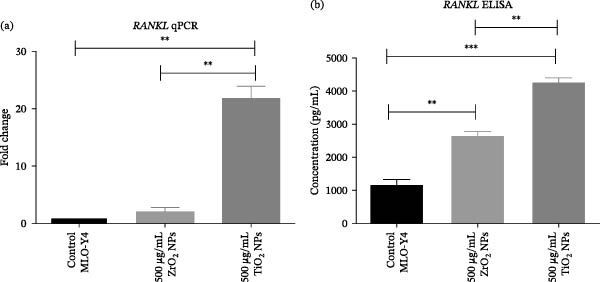
Expression of *RANKL* gene and protein by qPCR and ELISA at 72 h. (a) Histogram representing qPCR quantification of the total *RANKL* gene expression by MLO‐Y4 cells treated with 500 µg/mL ZrO_2_‐NPs and TiO_2_‐NPs. (b) Histogram representing ELISA measurement of total RANKL protein released by MLO‐Y4 cells treated with 500 µg/mL ZrO_2_‐NPs and TiO_2_‐NPs. Data represent the mean ± SD of three (*n* = 3) biological replicates ( ^∗∗^ *p* < 0.01,  ^∗∗∗^ *p* < 0.001).

At the gene level, *RANKL* expression in MLO‐Y4 cells was significantly upregulated following treatment with 500 µg/mL TiO_2_‐NPs (21.76‐fold ± 1.51; *p* < 0.01) compared to the control group. However, no significant difference was observed in the ZrO_2_‐NPs‐treated group (2‐fold ± 0.53) relative to the control. Intergroup analysis further confirmed that *RANKL* gene expression was significantly higher in the TiO_2_‐NPs‐treated group than in the ZrO_2_‐NPs‐treated group (*p* < 0.01) (Figure 5a).

At the protein level, RANKL release was significantly increased in MLO‐Y4 cells treated with both 500 µg/mL ZrO_2_‐NPs (2665 ± 81.25 pg/mL; *p*  < 0.01) and 500 µg/mL TiO_2_‐NPs (4282.25 ± 83.125 pg/mL; *p* < 0.001) compared to the control group (1185 ± 98.75 pg/mL). Furthermore, similar to SOST release, RANKL protein levels were significantly higher in the TiO_2_‐NPs‐treated group than in the ZrO_2_‐NPs‐treated group (*p* < 0.01) (Figure 5b).

### 3.5. *OPG* Gene Expression and Release by MLO‐Y4 Osteocytes Exposed to ZrO_2_ and TiO_2_‐NPs

**Figure 6:**
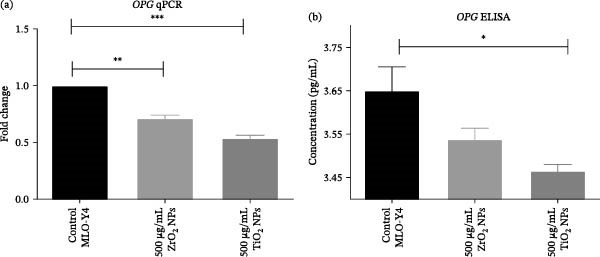
Expression of *OPG* gene and protein by qPCR and ELISA at 72 h. (a) Histogram representing qPCR quantification of the total *OPG* gene expression by MLO‐Y4 cells exposed to 500 µg/mL ZrO_2_‐NPs and TiO_2_‐NPs. (b) Histogram representing ELISA measurement of the total OPG protein released by MLO‐Y4 cells treated with 500 µg/mL ZrO_2_‐NPs and TiO_2_‐NPs. Data represent the mean ± SD of three (*n* = 3) biological replicates ( ^∗^ *p* < 0.05,  ^∗∗^ *p* < 0.01,  ^∗∗∗^ *p* < 0.001).

Both OPG gene expression and protein release followed a consistent trend. At the gene level, OPG expression was significantly downregulated in MLO‐Y4 cells treated with 500 µg/mL ZrO_2_‐NPs (0.71‐fold ± 0.02; *p* < 0.01) and 500 µg/mL TiO_2_‐NPs (0.54‐fold ± 0.02; *p* < 0.001) compared to the control. However, no significant difference was observed between the TiO_2_‐NPs and ZrO_2_‐NPs‐treated groups (Figure 6a).

Similarly, at the protein level, OPG release was significantly reduced following treatment with TiO_2_‐NPs (3.46 pg/mL; *p* < 0.05), while ZrO_2_‐NPs (3.53 pg/mL); (*p* > 0.05) had no significant effect compared to the control (3.65 pg/mL). Intergroup analysis confirmed no significant difference in OPG release between TiO_2_‐NPs and ZrO_2_‐NPs groups (Figure 6b).

## 4. Discussion

Titanium‐based materials have been widely used as endogenous implants in orthopedic and dental surgery over the past four decades, while ZrO_2_‐based implants have recently gained popularity in the field through their applications as biosensors, drug delivery systems, and implant dentistry. The present study compared the effects of ZrO_2_‐NPs and TiO_2_‐NPs on osteocyte viability, apoptosis, and expression of metabolic bone biomarkers. Our findings highlight distinct cytotoxic and osteogenic regulatory responses induced by both types of nanoparticles in MLO‐Y4 osteocytes, offering novel insights into their potential impact on bone homeostasis.

Cell morphology studies revealed that at higher concentrations of both the nanoparticles (500 and 1000 µg/mL), osteocytes exhibited significant morphological alterations, including rounding and loss of stellar morphology. This observation was further confirmed by viability assays, which confirmed a dose‐dependent response, with both types of nanoparticles reducing cell viability at 500 and 1000 µg/mL. While ZrO_2_‐NPs increased cell proliferation at lower concentrations, TiO_2_‐NPs caused a significant reduction in the viability of osteocytes at 100 µg/mL as early as 24 h.

To further explore and comprehend the mechanisms underlying osteocyte cytotoxicity, the current study performed apoptosis and necrosis assays, which also showed a similar dose‐dependent cytotoxic response to both ZrO_2_‐NPs and TiO_2_‐NPs. While 100 µg/mL ZrO_2_‐NPs exhibited minimal apoptosis and necrosis, TiO_2_‐NPs at the same concentration induced significant cell death. At 500 µg/mL, both types of nanoparticles markedly increased apoptosis, with TiO_2_‐NPs eliciting higher apoptotic levels. Necrosis was also significantly elevated, though TiO_2_‐NPs induced greater necrosis at 100 µg/mL, whereas ZrO_2_‐NPs caused higher necrotic rates at 500 µg/mL. In this study, exposure to high concentrations of TiO_2_ and ZrO_2_ NPs led to distinct patterns of cell death, with TiO_2_ NPs associated with a relatively higher proportion of apoptotic cells and ZrO_2_ NPs with a relatively higher proportion of necrotic cells at 500 µg/mL. Although the underlying mechanisms were not directly investigated here, these divergent apoptosis/necrosis profiles suggest that TiO_2_ and ZrO_2_ NPs may differentially engage cell death pathways. Based on previous nanoparticle toxicology studies, it is plausible that differences in physicochemical properties (e.g., surface reactivity, charge, and aggregation behavior) could influence the balance between programmed cell death and loss of membrane integrity [20, 21]. Other factors that may contribute but were not assessed in the present work include nanoparticle‐membrane interactions and cellular internalization dynamics, which can modulate subcellular localization and the nature of stress signals perceived by the cell. These considerations remain speculative in the context of our data, and future studies incorporating measurements of oxidative stress, membrane damage, and uptake kinetics will be required to delineate the precise mechanisms.

Nanoparticle physicochemical properties such as hydrodynamic size, agglomeration state, and surface charge strongly influence cellular uptake and biological responses. Recent studies emphasize the need for thorough characterization when interpreting nanoparticle‐cell interactions [22, 23]. In our study, both TiO_2_‐ and ZrO_2_‐NPs were commercially sourced from the same manufacturer, and TiO_2_‐NPs had been previously characterized under identical dispersion conditions. Although both nanoparticle types were prepared using the same medium and sonication protocol to minimize variability, we acknowledge that parallel hydrodynamic characterization of ZrO_2_‐NPs would further strengthen direct comparisons. Future work will include full side‐by‐side characterization to better clarify how specific physicochemical properties influence osteocyte responses. These results align with previous studies demonstrating that TiO_2_/ZrO_2_‐NPs induced apoptotic and necrotic responses reported in various cells, like human keratinocyte cells [24], HeLa cells [25], and osteoblast‐like cells [26].

Based on these findings, in the current study, a 500 µg/mL concentration for both types of nanoparticles was chosen for downstream experiments as it provided an optimal balance between cellular stress and viability. While viability significantly declined at this concentration, it remained above 50%, ensuring sufficient cell survival. The concentrations used in this study are relatively high compared with systemic metal levels typically detected in blood or saliva, which are usually in the ng/mL‐µg/L range. Clinical and experimental studies, however, indicate that corrosion and tribocorrosion of dental implants lead to substantial accumulation of titanium particles and ions in peri‐implant tissues, particularly in inflamed sites, with local tissue concentrations reported in the order of 100–300 ppm (i.e., high µg/mL range), whereas distant organs exhibit much lower levels. Therefore, our in vitro concentrations may be viewed as representing local “worst‐case” peri‐implant microenvironments at the immediate implant‐tissue interface, rather than typical systemic exposure conditions, which similarly reported concentration‑dependent biological effects of titania NPs and highlighted that upper‑range in vitro doses can approximate localized peri‑implant exposure conditions [27, 28]. Their findings reinforce the use of high, subcytotoxic concentrations, such as the 500 µg/mL applied in our study to model “worst‑case” microenvironments where nanoparticle accumulation is greatest.

The MLO‑Y4 cell line is an accepted model for nanoparticle–osteocyte interactions because it recapitulates key features of early osteocytes, including dendritic processes, expression of osteocyte‑associated markers, and the ability to communicate with and modulate osteoclasts and osteoblasts. However, important limitations must be acknowledged: MLO‑Y4 cells are of murine origin and thus may not fully reflect human osteocyte biology; they represent a partial/early osteocyte phenotype with low expression of late osteocyte markers such as DMP1 and SOST; and, like most in vitro systems, they are cultured in 2D, which cannot fully reproduce the three‑dimensional lacuno‑canalicular network and mechanical microenvironment present in vivo. These factors should be considered when extrapolating our nanoparticle–osteocyte findings to the human bone environment in vivo [29].

While osteocyte apoptosis under homeostatic conditions is in an optimal state of balance, tightly controlled by proapoptotic and antiapoptotic mechanisms, pathological osteocyte apoptosis may upregulate SOST and SOST release, leading to resorption‐related bone disease [30]. Furthermore, other workers have demonstrated that SOST inhibits the Wnt/β‐catenin signaling pathway by binding the LRP5/6 and Frizzled coreceptors on osteoblasts, reducing bone formation by inhibiting osteoblast differentiation and activity [31]. In addition to its antianabolic effects, SOST has also been shown to promote osteoclast activity by enhancing RANKL‐mediated signaling in osteocytes, as demonstrated by Cameron et al. [32], further supporting its central role in coordinating bone resorption. This study has also demonstrated that induction of pathological apoptosis following exposure to both ZrO_2_‐NPs and TiO_2_‐NPs leads to a significant increase in SOST gene expression and SOST protein release in osteocyte‐like cells, with TiO_2_‐NPs inducing significantly higher SOST levels. The magnitude of this effect was substantial, with TiO_2_‐NPs eliciting an 18‐fold increase in mRNA expression compared to a 10‐fold increase for ZrO_2_‐NPs. While protein levels peaked at 24 h, they returned to baseline by 72 h despite sustained mRNA upregulation. This discrepancy likely reflects rapid protein turnover, extracellular matrix sequestration, or a transient secretory burst often seen in nanoparticle‐induced stress responses. Time‑dependent nanoparticle‐cell interactions may also explain this transient pattern. Cameron et al. [32] demonstrated that nanoparticle‑induced stress responses often peak early and diminish as cells undergo adaptive or compensatory changes. This framework supports our observation that SOST protein release was transient, with an early surge at 24 h, followed by normalization at 72 h despite continued SOST transcription. Such divergence between mRNA and protein levels is consistent with rapid protein turnover, extracellular matrix sequestration, or feedback‑regulated suppression of secretion.

Notably, SOST upregulates RANKL expression and downregulates OPG production in osteocytes [32, 33], and this is linked to an increased RANKL/OPG ratio, promoting osteoclastogenesis. In the current study, TiO_2_‐NPs significantly increased RANKL expression while reducing OPG, aligning with previous reports on titanium dioxide particle‐induced osteocyte signaling [34]. The biological relevance of this shift is highlighted by the 22‐fold upregulation of RANKL gene expression in TiO_2_‐treated cells, whereas ZrO_2_‐NPs induced only a nonsignificant 2‐fold change. Although ZrO_2_‐NPs followed a similar trend in the current study, their impact on SOST expression, RANKL, and OPG release was notably lower than that of TiO_2_‐NPs, suggesting a comparatively less intense effect of ZrO_2_‐NPs on bone resorption. The absolute concentrations of RANKL protein were also nearly 1.6 times higher in the TiO_2_ group (4282 pg/mL) than in the ZrO_2_ group (2665 pg/mL), further supporting the less disruptive profile of ZrO_2_. Although these molecular changes indicate a shift toward a pro‑osteoclastogenic signaling environment, they do not confirm that osteoclast differentiation actually occurred. As emphasized by Diogo et al. [35], functional validation, such as osteoclast precursor cultures or conditioned‑media assays, is essential to determine whether altered RANKL/OPG signaling translates into increased osteoclast formation. This represents a limitation of the present study, and future work will incorporate functional coculture or conditioned‑media models to directly assess osteoclastogenesis.

While the current study provides significant insights, several limitations must be acknowledged. First, the use of a 2D in vitro culture system and a murine‐derived cell line may not fully recapitulate the complex 3D lacuno‐canalicular network or the specific biology of human osteocytes. Second, this model lacked mechanical loading and an inflammatory milieu, both of which are critical factors in peri‐implant bone remodeling in vivo. Third, the nanoparticle doses used (500 µg/mL) represent a worst‐case local accumulation scenario, and there might be potential confounding by endotoxin contamination. Furthermore, this study utilized a single osteocyte model without coculture with osteoclast precursors; therefore, the observed shifts in the RANKL/OPG axis provide a molecular rationale but not a functional confirmation of increased osteoclastogenesis. Future studies incorporating 3D scaffolds and functional coculture assays are required to validate these findings. In addition, the simplified 2D murine osteocyte model limits direct in vivo translation as it lacks the human osteocyte phenotype, 3D lacuno‑canalicular structure, and mechanical loading that shape osteocyte signaling. As noted by Sequeira et al. [36], biomaterial‐cell interactions must be interpreted within the context of the native mechanical and structural microenvironment, which this model cannot reproduce.

This study indicates that implant‑derived nanoparticles can influence osteocyte signaling toward a more catabolic profile. Under the specific in vitro conditions tested, ZrO_2_‑NPs elicited a relatively lower osteocyte catabolic response than TiO_2_‑NPs, without implying broader clinical superiority. The findings highlight that osteocyte‑mediated pathways are important in evaluating biomaterial compatibility and underscore the need for future studies incorporating 3D osteocyte models, mechanical loading, nanoparticle‑internalization imaging, oxidative stress responses, and in vivo validation to clarify the translational relevance of nanoparticle and osteocyte interactions for peri‑implant bone health.

## Author Contributions

Mustafa Hayder Matouk contributed to conceptualization, data curation, formal analysis, investigation, methodology, software, visualization, and writing – original draft. K. G. Aghila Rani contributed to data curation, formal analysis, investigation, methodology, visualization, project administration, supervision, and writing – review and editing. Waad Kheder contributed to data curation, visualization, supervision, project administration, and writing – review and editing. Asmaa Ismail contributed to visualization, project administration, methodology, visualization, and writing – review and editing. A. R. Samsudin contributed to visualization, project administration, supervision, visualization, and writing – review and editing. Sausan AlKawas contributed to funding acquisition, resources, supervision, validation, visualization, and writing – review and editing. Sausan AlKawas (corresponding author) had full access to all of the data in this study and takes complete responsibility for the integrity of the data and the accuracy of the data analysis.

## Funding

The funding for the study was supported by the University of Sharjah Graduate Studies Research Grant.

## Disclosure

All authors have read and approved the final version of the manuscript.

## Conflicts of Interest

The authors declare no conflicts of interest.

## Supporting Information

Additional supporting information can be found online in the Supporting Information section.

## Supporting information


**Supporting Information** Table S1: Physicochemical Profiling of TiO_2_ NPs.

## Data Availability

The data that support the findings of this study are available from the corresponding author upon reasonable request.
