# Supplementary material for: A Comparative Study on the Influence of Zirconia and Titanium Nanoparticles on the Functionality of Osteocytes In Vitro
Source: Int J Dent. 2026 Jun 14;2026:6486868. doi: 10.1155/ijod/6486868 (PMC13266283; doi:10.1155/ijod/6486868)
Supplement: Supplementary file 1 — Supporting Information Table S1: Physicochemical Profiling of TiO2 NPs. [file IJOD-2026-6486868-s001.docx]

**Supplementary Table 1**

Physicochemical Profiling of TiO₂ NPs

| **Particle Type** | **Hydrodynamic Size (nm)** | **PDI** | **Zeta Potential (mV)** |
| --- | --- | --- | --- |
| TiO₂ NPs | 341.03 ± 36.82 | 0.29 ± 0.11 | −7.4 ± 0.93 |

*Kheder et al., 2021*
